# Systematic review and meta-analysis of multiparametric MRI clear cell likelihood scores for classification of small renal masses

**DOI:** 10.3389/fonc.2022.1004502

**Published:** 2022-10-26

**Authors:** Jun Tian, Feixiang Teng, Hongtao Xu, Dongliang Zhang, Yinxiu Chi, Hu Zhang

**Affiliations:** ^1^ Department of Basic Medication, Jiangsu Vocational College of Medicine, Yancheng, China; ^2^ Department of Medical Imaging, Jiangsu Vocational College of Medicine, Yancheng, China

**Keywords:** clear cell likelihood score, MRI, classification, meta-analysis, small renal mass

## Abstract

**Purpose:**

To systematically assess the multiparametric MRI clear cell likelihood score (ccLS) algorithm for the classification of small renal masses (SRM).

**Methods:**

We conducted an electronic literature search on Web of Science, MEDLINE (Ovid and PubMed), Cochrane Library, EMBASE, and Google Scholar to identify relevant articles from 2017 up to June 30, 2022. We included studies reporting the diagnostic performance of the ccLS for characterization of solid SRM. The bivariate model and hierarchical summary receiver operating characteristic (HSROC) model were used to pool sensitivity, specificity, positive likelihood ratio (LR+), negative likelihood ratio (LR−), and diagnostic odds ratio (DOR). The quality evaluation was performed with the Quality Assessment of Diagnostic Accuracy Studies-2 tool.

**Results:**

A total of 6 studies with 825 renal masses (785 patients) were included in the current meta-analysis. The pooled sensitivity and specificity for cT1a renal masses were 0.80 (95% CI 0.75–0.85) and 0.74 (95% CI 0.65–0.81) at the threshold of ccLS ≥4, the pooled LR+, LR−, and DOR were 3.04 (95% CI 2.34-3.95), 0.27 (95% CI 0.22–0.33), and 11.4 (95% CI 8.2-15.9), respectively. The area under the HSROC curve was 0.84 (95% CI 0.81–0.87). For all cT1 renal masses, the pooled sensitivity and specificity were 0.80 (95% CI 0.74–0.85) and 0.76 (95% CI 0.67–0.83).

**Conclusions:**

The ccLS had moderate to high accuracy for identifying ccRCC from other RCC subtypes and with a moderate inter-reader agreement. However, its diagnostic performance remain needs multi-center, large cohort studies to validate in the future.

## Introduction

Over the past couple of decades, the incidence of renal cell carcinoma (RCC) has steadily increased in the United States and worldwide, in which cross-sectional imaging is play an important role (1–4). Indeed, as many as 70% of RCCs are detected incidentally for unrelated medical conditions (5). Higher detection of small renal lesions results in at least 80% increase in the number of surgical resections but does not bring considerable benefit to cancer-specific mortality at the population level (6, 7). Additionally, many renal masses exhibit an indolent behavior or grow very slowly and need no intervention (8, 9). Renal mass biopsies are recommended by several groups to facilitate personalized management; however, its nondiagnostic is up to 20% and not feasible in all patients (10). Thus, using non-invasive imaging examinations such as MRI and CT represents an alternative to biopsy to assist location, staging, and management of renal masses (1, 11, 12).

For cystic renal masses, the Bosniak classification provides standardized risk stratification and has been widely utilized in clinical practice for decades (13, 14). With respect to small solid renal masses, however, there is no widely accepted standardized risk stratification up to date, even though many studies demonstrated that imaging techniques such as US, CT, and MRI may play an important role in prediction of tumor histologic findings (15). Clear cell RCC (ccRCC) is the most common subtype of various RCC, accounting for more than half of cases and associated with worse outcome as compared to papillary and chromophobe tumors (16, 17). In addition, ccRCC is the most common cause of disease progression and metastasis in patients under active surveillance based on the combination of characteristics (18). In 2017, Canvasser et al. proposed the five-category Likert scale named ccLS to evaluate whether an SRM being a ccRCC (from 1 point=very unlikely to 5 points=very likely) (19). To date, several published studies have reported that this scoring system performed well in clinical practice; however, this algorithm has not been systematically assessed. Therefore, the purpose of this study was to evaluate the overall performance of the ccLS algorithm for the classification of ccRCC.

## Methods

This meta-analysis and systematic review was conducted according to the Preferred Reporting Items for Systematic Reviews and Meta-Analysis (PRISMA) statement, with a predefined review and data extraction protocol (20). The primary outcome of our study was the diagnostic accuracy of the ccLS for identifying the cT1a (≤4 cm) solid renal masses. Additionally, considering that some studies applied the ccLS to cT1b (>4 cm and ≤7 cm) masses, we would assess the diagnostic performance of this algorithm for all cT1 (≤7 cm) renal masses.

### Search strategy and selection criteria

We conducted a systematic search of PubMed, EMBASE, Cochrane Library, Web of Science, and Google Scholar online scientific publication databases to identify articles published between January 2017 and June 2022, by using Medical Subject Headings (MeSH) and restricted language to English. The following terms and synonyms was used for literature searching: ([kidney] OR (renal) OR (nephron)] AND [(cancer) OR (mass*) OR (lesion)] AND ([ccLS] OR [clear cell likelihood score]). We supplemented our searches by manually screening the bibliographies of reviews and eligible articles. Two reviewers (*T.J.* and *T.F.X.*) evaluated the results of the literature search independently, and discrepancies were resolved by discussion with a third reviewer (*Z.H.*).

### Inclusion and exclusion criteria

We included studies that satisfied all of the following criteria: 1) using the ccLS for characterization of ccRCC; 2) providing sufficient details for reconstruction of 2×2 contingency tables for determination of the diagnostic accuracy; and 3) with biopsy or surgical pathological results as the reference standard. We excluded studies that met any of the following criteria: 1) not using the ccLS but other scoring systems or subjective assessment; 2) case reports or case series involving less than 20 participants; 3) with insufficient data to assess the diagnostic performance; 4) meta-analyses, guidelines, editorials, reviews, and letters; and 5) with partially overlapping patient populations.

### Data extraction and quality assessment

We extracted the following information from included studies with a standardized form: 1) demographic and clinical characteristics such as sample size of patients and masses, patient age, male-to-female ratio, and tumor size; 2) study characteristics such as authors, study design, year of publication, country and period of the study conducted, number of readers and their experience, inter-reader agreement, blinding to final results, and reference standard; and 3) technical characteristics such as MRI sequences and magnetic field strength. We employed the Quality Assessment of Diagnostic Accuracy Studies–2 to evaluate the study quality (21), in which the risk of bias for each study was assessed according to four domains: patient selection, method of the index test, reference standard, and flow and timing. Data extraction and quality assessment was carried out by two reviewers (*T.J.* and *T.F.X.*) independently.

### Data synthesis and statistical analysis

The bivariate model and HSROC model were used to pool the summary estimate of sensitivity, specificity, LR+, LR−, DOR, and their 95% confidence intervals (CIs) (22, 23). In addition, we constructed the forest plots and HSROC curve to graphically present the results. The Deeks’ funnel plot was used to evaluate the publication bias, and the Deeks’ asymmetry test was used to decide statistical significance (24). The degree of heterogeneity between studies was measured with Cochran *Q* statistics and Higgins *I* ^2^: for value of 0%-40%, not important; for value of 30%-60%, moderate; for value of 50%-90%, substantial; for value of 75%-100%, considerable (25). The “metandi” and “midas” modules in STATA 16.0 (StataCorp, Texas, USA) were used for all analyses, with a *P <*0.05 indicating statistically significant.

## Results

### Literature search and data extraction

**Figure 1:**
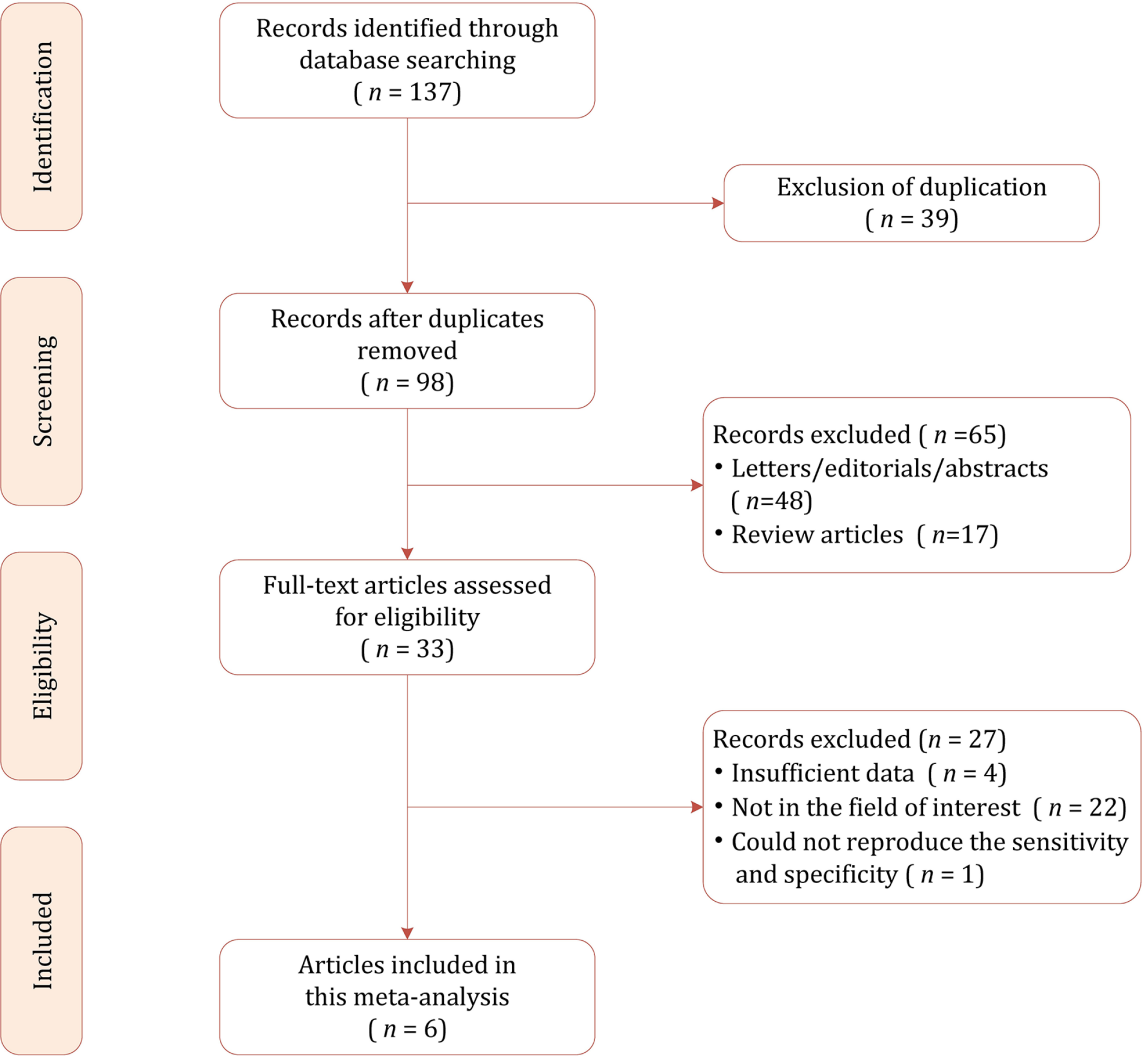
Study selection process for this systematic review and meta-analysis.

The flow chart of the literature selection process is presented in **Figure 1**. Our search strategy yielded 137 results initially, of which 39 were removed due to duplicates. After screening the titles and abstracts, a total of 65 results were excluded. Full-text reviewing was performed among the remaining 33 potential results and 27 were excluded for insufficient data (*n*=5), not in the field of interest (*n*=22). Ultimately, a total of 6 studies involving 785 patients were included in this meta-analysis (19, 26–30).

### Characteristics of the included studies

The detailed demographic and study characteristics are summarized in **Tables 1** and **2**. Regarding study design, nearly all studies were retrospective, and the sample size of the study population ranged from 57 to 241 patients. The mean age for patients ranged from 57 to 67 years, with an average tumor size of 24-38 mm. The proportion of ccRCC among studies was 41.7%-65.7%. Regarding the number of radiologists, 1 study reported that images were interpreted by only one reader (28), whereas in the remaining 5 studies images were interpreted by at least 3 readers. The reported radiologists’ experience ranged from 1 to 30 years, with inter-reader agreement measured with kappa value of 0.53-0.65. Regarding cutoff values, 3 studies reported that the results were derived from the threshold of ccLS ≥4 (26, 28, 30), whereas the remaining 3 studies reported results from both ccLS ≥3 and ccLS≥4 (19, 27, 29). Concerning technique characteristics, nearly all studies reported that the images acquired from 1.5T or 3.0 T MRI; however, in one study the field strength was not reported (28). As for MRI protocol, only 3 studies used all sequences of T1, T2, dynamic contrast-enhanced (DCE), and diffusion-weighted imaging (DWI) (27, 29, 30). Concerning the reference standard, surgical resection pathological results were used in 4 studies, in the remaining 2 studies the biopsy results also were used in case of pathological results were not available (26, 27).

**Table 1:** Demographic Characteristics of Included Studies.

Study	Country	Year	No. of patients	No. of lesions	Type of masses	No. ofccRCC	Gender(M/F)	Age(year, mean±SD/median)	Tumor Size(cm, mean±SD/median)
**Canvasser et al.**	USA	2017	110	121	cT1a	61	61/39	57±14	2.4±0.8
**Dunn et al.**	Canada	2022	102	108	cT1a/cT1b	45	67/53	56.9±12.8	3.0±1.3
**Johnson et al.**	USA	2019	57	63	cT1a	35	38/19	61.7±14.9	2.7±0.7
**Morgan et al.**	USA	2021	70	70	cT1	66	45/25	67/61-72	3.8/2.8-4.8
**Schieda et al.**	USA/Canada	2022	241	250	cT1a	119	174/76	60±13	2.5±0.8
**Steinberg et al.**	USA	2020	204	213	cT1a/cT1b	183	110/94	59±13	2.7±0.8

ccRCC, clear cell renal cell carcinoma; SD, standard deviation.

**Table 2:** Study Characteristics of Included Studies.

FirstAuthor	StudyDesign	Study Period	No. ofReaders	Experience(Years)	Magnet Field Strength	Blinded	MRISequence	CutoffValue	*κ* Value	Reference
**Canvasser et al.**	Retrospective	2011.12- 2015.07	7	1-15	1.5 T/3.0 T	Yes	T1/T2/DCE	≥3/≥4	0.53/0.38-0.64	Histological
**Dunn et al.**	Retrospective	2013.01-2018.02	3	7-12	1.5 T	Yes	T1/T2/DCE	≥4	0.65	Biopsy/ Histological
**Johnson et al.**	Prospective	2016.06-2018.04	14	NA	1.5 T/3.0 T	Yes	T1/T2/DCE/DWI	≥3/≥4	NA	Biopsy/ Histological
**Morgan et al.**	Retrospective	2016.04-2020.02	1	NA	NA	Yes	NA	≥4	NA	Histological
**Schieda et al.**	Retrospective	2012.12-2019.12	10	5-30	1.5 T/3.0 T	Yes	T1/T2/DCE/DWI	≥3/≥4	0.58/0.42-0.75	Histological
**Steinberg et al.**	Retrospective	2016.06-2019.11	16	NA	1.5 T/3.0 T	Yes	T1/T2/DCE/DWI	≥4	NA	Histological

DCE, dynamic contrast enhanced; DWI, diffusion weighted imaging; NA, not available; T1, T1 weighted imaging; T2, T2 weighted imaging.

### Quality assessment

**Figure 2:**
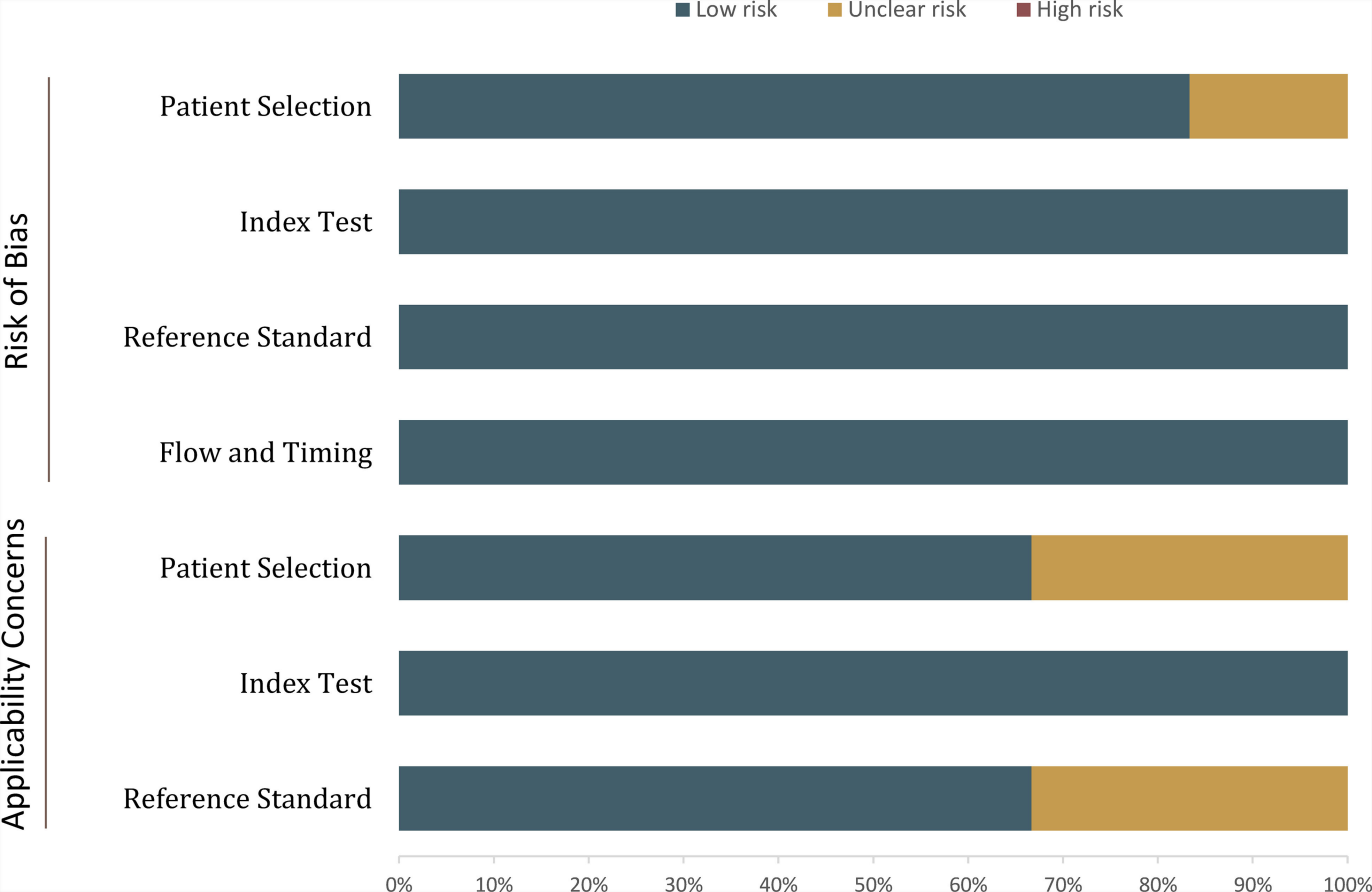
Grouped bar charts show the risk of bias and concerns for applicability of included studies.

The overall quality assessment of the included studies was high. With respect to the type of renal masses, 1 study included all of the cT1 renal masses, thus was assigned as high risk of bias. In more than half studies, the analysis was restricted to masses with confirmed pathological results, which may lead to selection and verification biases as those masses under surveillance and did not undergo histological confirmation were not included. For the reference standard domain, in 2 studies the biopsy results were also used as the reference standard. Concerning the flow and timing domain, all included studies were assigned low risk of bias, detailed quality assessment is presented in **Figure 2**.

### Diagnostic performance of the ccLS for renal masses

**Figure 3:**
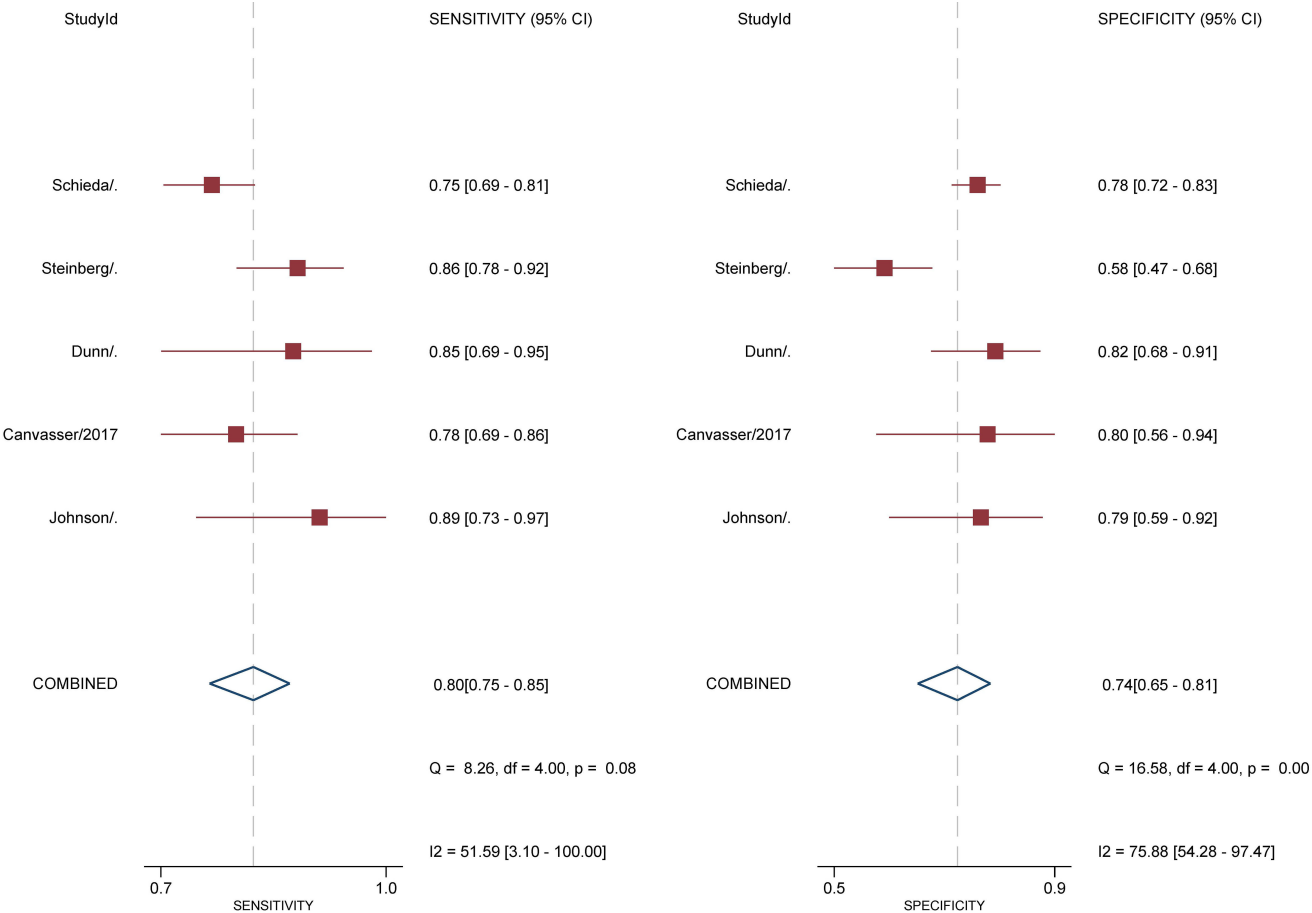
Coupled forest plot of pooled sensitivity and specificity. Numbers are pooled estimates with 95% CI in parentheses. Corresponding heterogeneity statistics are provided at bottom right corners. Horizontal lines indicate 95% confidence intervals.

**Figure 4:**
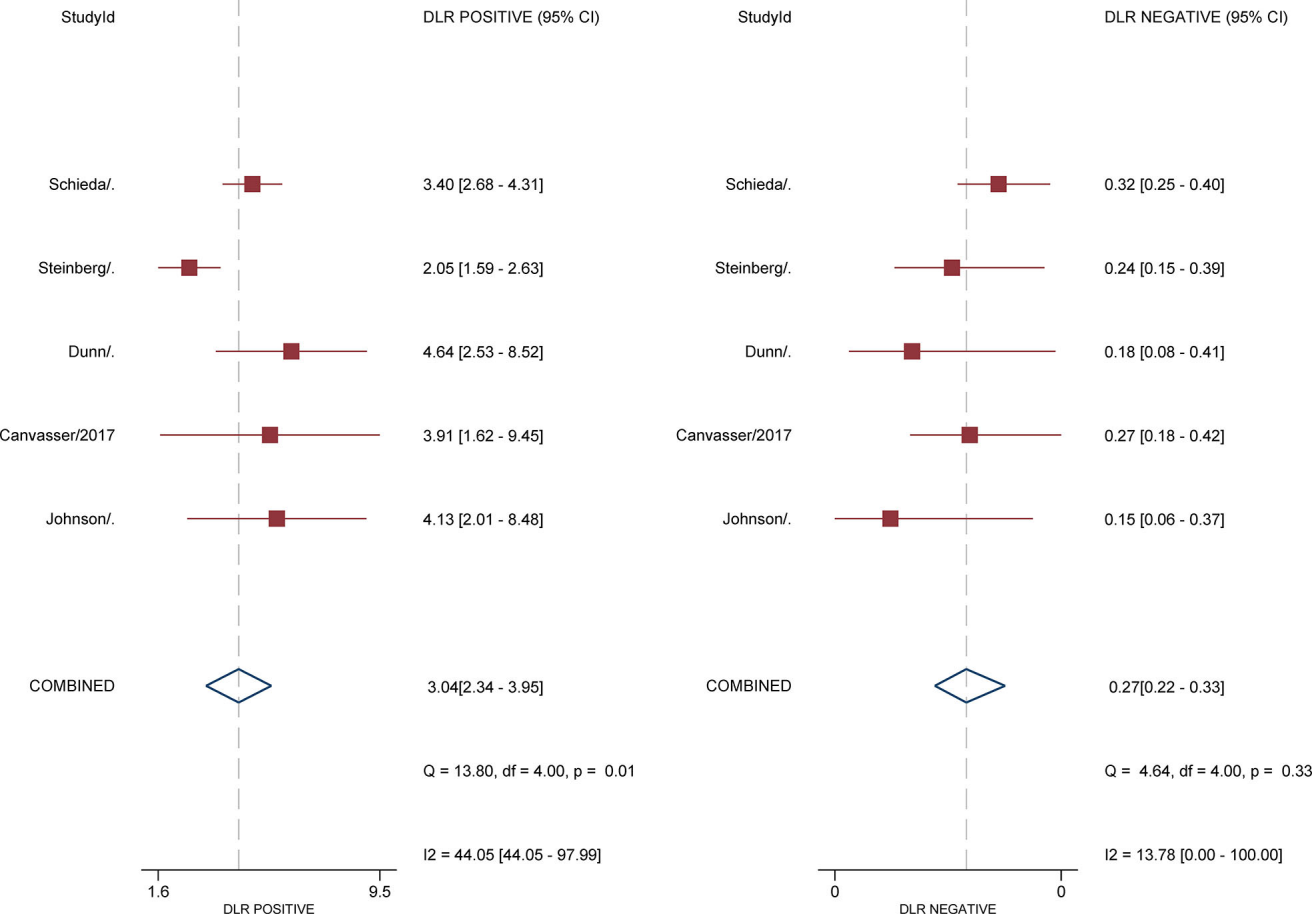
Coupled forest plot of pooled negative and positive likelihood ratios.

**Figure 5:**
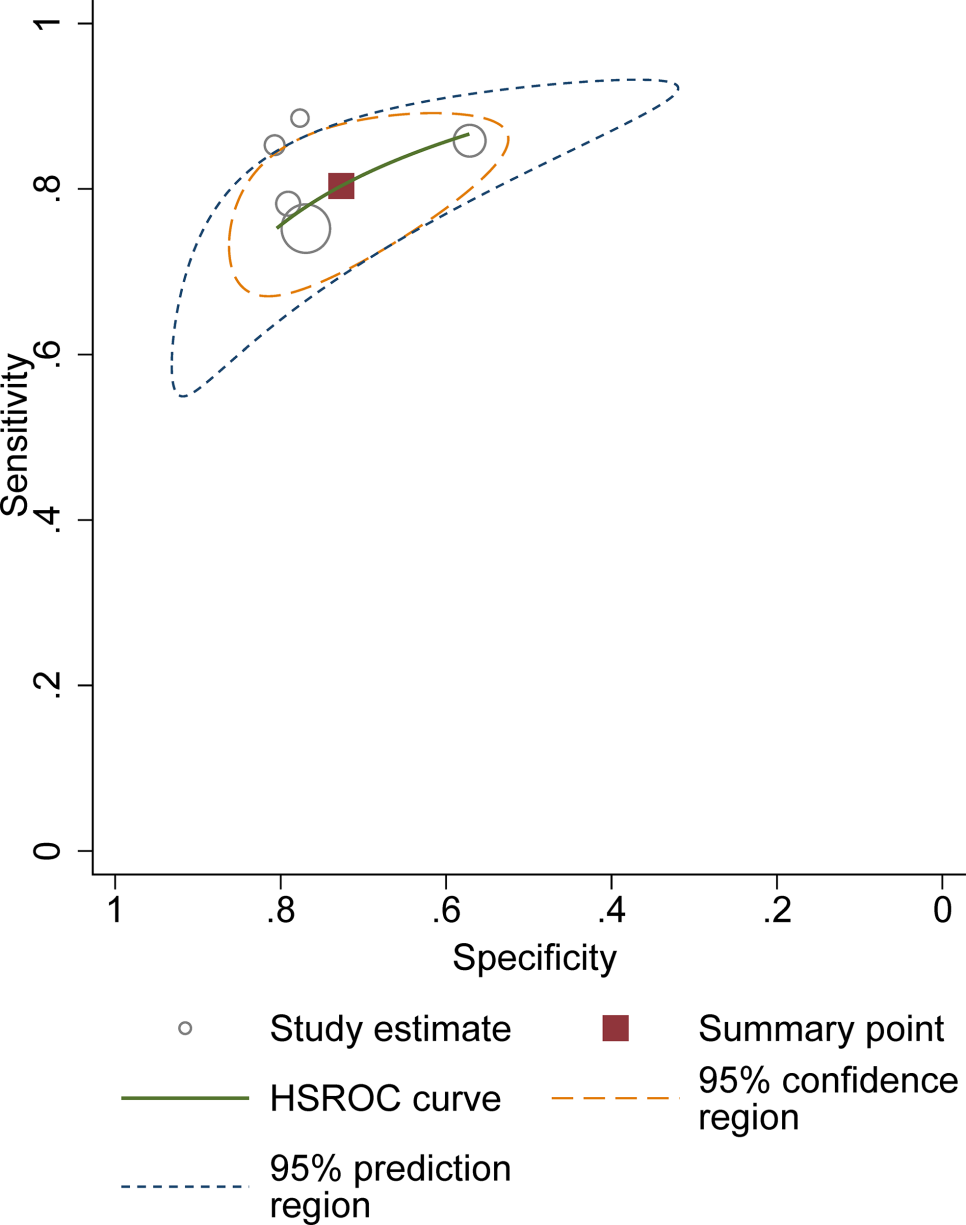
Hierarchic summary receiver operating characteristic plots with summary point and 95% confidence area for the overall.

**Figure 6:**
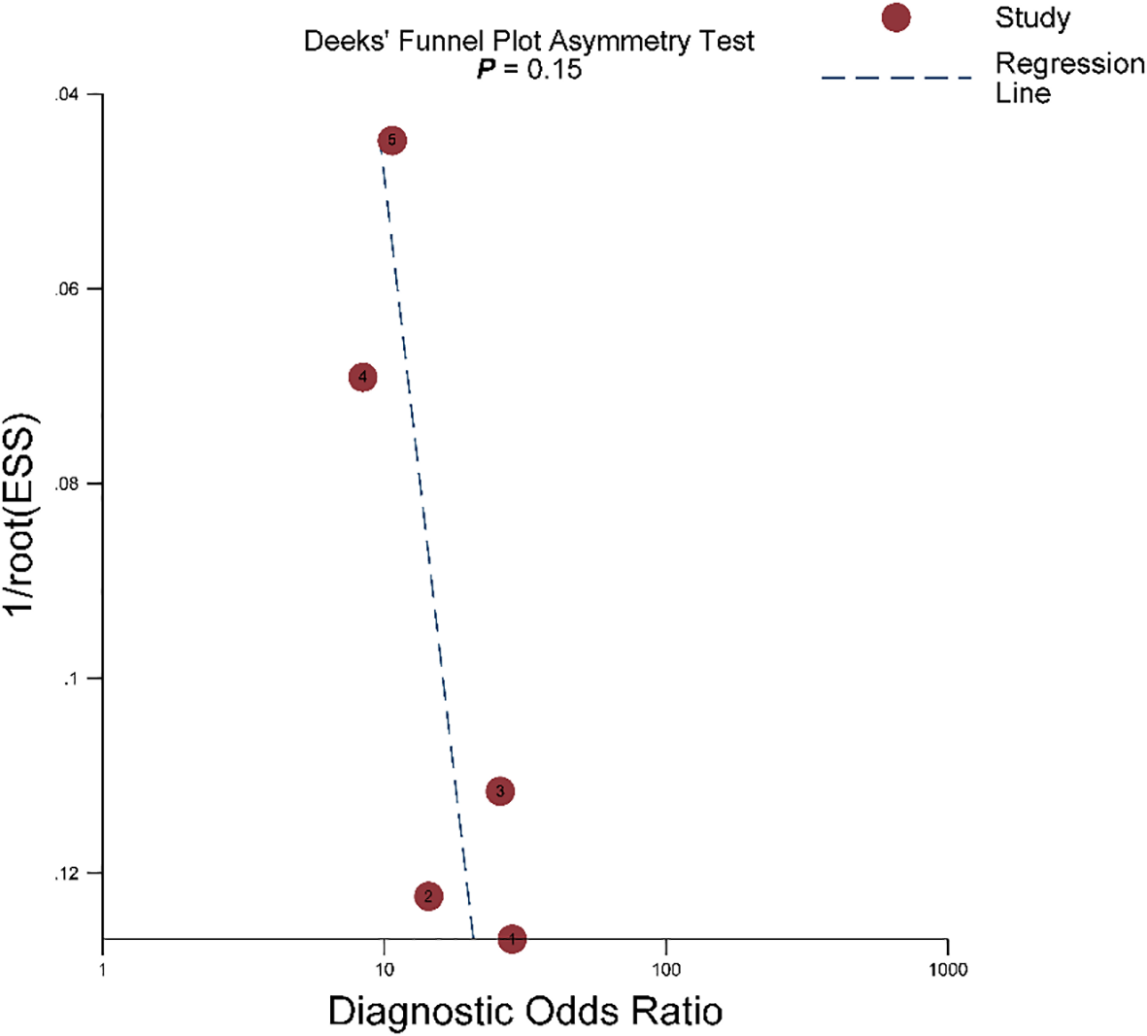
The Deeks’ funnel plot.

For 5 studies using the ccLS for risk stratification of cT1a ccRCC, the sensitivity and specificity were 0.75-0.89 and 0.58-0.82 for individual studies. The summary estimates of sensitivity and specificity for cT1a renal masses were 0.80 (95% CI 0.75-0.85) and 0.74 (95% CI 0.65-0.81), respectively, with the area under HSROC of 0.84 (95% CI 0.81-0.87). Coupled forest plots are presented in **Figure 3**. The pooled LR+, LR−, and DOR were 3.04 (95% CI 2.34-3.95), 0.27 (95% CI 0.22–0.33), and 11.4 (95% CI 8.2-15.9), respectively (**Figure 4**). The *Q* test revealed substantial heterogeneity presented throughout studies (P<0.05), and Higgins *I* ^2^ statistics also indicated substantial heterogeneity in terms of both sensitivity (*I ^2 =^* 51.6%) and specificity (*I* ^2 =^ 75.9%). In the HSROC curve, a large difference between the 95% confidence region and the 95% prediction region suggested substantial heterogeneity among studies (**Figure 5**). The Deeks funnel plot is presented in **Figure 6**, the P value of 0.15 for the slope coefficient indicated that the likelihood of publication bias was not statistically significant.

In the light of 3 studies providing the results of using the ccLS for stratification of cT1b renal masses, we then pooled the sensitivity and specificity of diagnostic accuracy for these lesions. The calculated summary estimates were comparable with cT1a masses, with pooled sensitivity and specificity of 0.83 (95% CI 0.71-0.89) and 0.78 (95% CI 0.58-0.91), respectively. For all cT1 renal masses (cT1a and cT1b), the pooled sensitivity and specificity were 0.80 (95% CI 0.74–0.85) and 0.76 (95% CI 0.67–0.83), with the calculated area under HSROC of 0.85 (0.82-0.88).

## Discussion

In this study, we systematically assessed the diagnostic performance of the ccLS for the classification of solid SRMs. Based on 6 studies, the pooled sensitivity and specificity at the threshold of ccLS ≥4 were 0.80 and 0.74, demonstrating moderate accuracy for cT1a renal masses. Despite the primary goal of ccLS is for classification of cT1a masses, some studies have applied it to cT1b masses. Our study suggested that the ccLS could also work well for all cT1 masses, with sensitivity and specificity of 0.80 and 0.76, respectively. Nevertheless, due to the small sample the diagnostic performance of the ccLS for all cT1 still needs large prospective multi-center studies to validate in the future. Reproducibility is critical for the standardized scoring system, as it relates to reducing the variability of interpretation between readers and improving the classification of solid SRMs. In the current meta-analysis, the included studies reported moderate inter-reader agreement between radiologists, with kappa value of 0.53-0.65.

At present, both the American Urologic Association and the American Society of Clinical Oncology recommend active surveillance as an initial management option for small renal masses, which is based on the fact that although approximately 80%-85% of small renal masses are malignant, only a minority showed the aggressive histologic features associated with disease progression and metastasis (31). Moreover, considering the patient morbidity and healthcare costs, active surveillance has been regarded as a viable management option for incidental small renal masses (18). Nevertheless, for ccRCC, the most common cause of disease progression and metastasis, active surveillance may occasionally yield unfavorable outcomes (16). Therefore, the need for better risk stratification strategies for indeterminate small solid renal masses is the main barrier to the wide acceptance of active surveillance in clinical practice (11). The emergence of the ccLS algorithm provides an encouraging start of standardization for solid renal mass, which represents the routine viewing approach from radiologists with less experience. According to the ccLS, assessment of the renal masses includes two primary steps: eligibility criteria, ensuring the absence of macroscopic fat and at least mild (defined as 25%) contrast enhancement; and major criteria, assessing signal on T2-weighted MRI scans, corticomedullary contrast-enhancement degree, and presence of intra-lesion microscopic fat (32). In addition to offer an algorithm for assessing the likelihood of renal masses being ccRCC, Rasmussen et al. found that SRMs assigned ccLS category 4–5 grew at a faster rate than those assigned ccLS category 1–2 or ccLS category 3, which could help avoid pathologic confirmation through biopsy in many patients before recommending active surveillance or other intervention (33).

As compared with CT, MRI provides excellent soft-tissue contrast to differentiate those solid from cystic masses when enhancement is questionable on CT, especially for lesions between 10 and 20 HU (34). Furthermore, with DCE and functional information such as DWI, MRI can provide specific information regarding tumor histology, to acquire multiple postcontrast phases routinely without ironizing radiation (35). Although using the ccLS algorithm yielded similar diagnostic accuracy to radiologists’ personal experience, this standardized workflow can assist radiologists with less experience to assess small SRMs with MRI (36). Moreover, the reported inter-reader agreement for this classification seemed moderate and comparable with other existing standardized scoring systems such as PI-RADS and TI-RADS (37, 38). Despite the ccLS has been assessed in several institutions, some improvements should be taken into account in the future version, e.g., the ccLS does not consider the other 2 RCC subtypes of papillary and chromophobe (32).

Our study has limitations that deserve mention. First, regarding study design, nearly all studies included were retrospective, which led to high risk of bias in terms of the patient selection domain. Nonetheless, considering that there was only one study of prospective, it was unfeasible to pool data for a single study. Second, considerable heterogeneity was observed between studies, which may lower the applicability of our study. Nevertheless, it is unfeasible to conduct meta-regression to explore the source of heterogeneity because merely 6 studies were included. However, the methodology for this meta-analysis was conducted strictly according to the Cochrane Collaboration guideline. Third, all studies did not report the results of experienced and inexperienced readers separately, therefore whether ccLS could work well among radiologists with less unknown is still unknown.

## Conclusion

Use of the ccLS algorithm could yield moderate sensitivity and specificity for evaluation of ccRCC, with a moderate inter-reader agreement. Considering the complex subtype of RCC, the ccLS offers an encouraging start of standardization for the assessment of ccRCC. However, its diagnostic performance needs multi-center large cohort studies to validate in the future.

## Data availability statement

The original contributions presented in the study are included in the article/supplementary material. Further inquiries can be directed to the corresponding author.

## Author contributions

All authors listed have made a substantial, direct, and intellectual contribution to the work, and approved it for publication.

## Conflict of interest

The authors declare that the research was conducted in the absence of any commercial or financial relationships that could be construed as a potential conflict of interest.

## Publisher’s note

All claims expressed in this article are solely those of the authors and do not necessarily represent those of their affiliated organizations, or those of the publisher, the editors and the reviewers. Any product that may be evaluated in this article, or claim that may be made by its manufacturer, is not guaranteed or endorsed by the publisher.
